# Supplementary figures and images for: Cryo-EM reconstructions of inhibitor-bound SMG1 kinase reveal an autoinhibitory state dependent on SMG8
Source: eLife. 2021 Oct 26;10:e72353. doi: 10.7554/eLife.72353 (PMC8592573; doi:10.7554/eLife.72353)

raw images for Figure 1 C

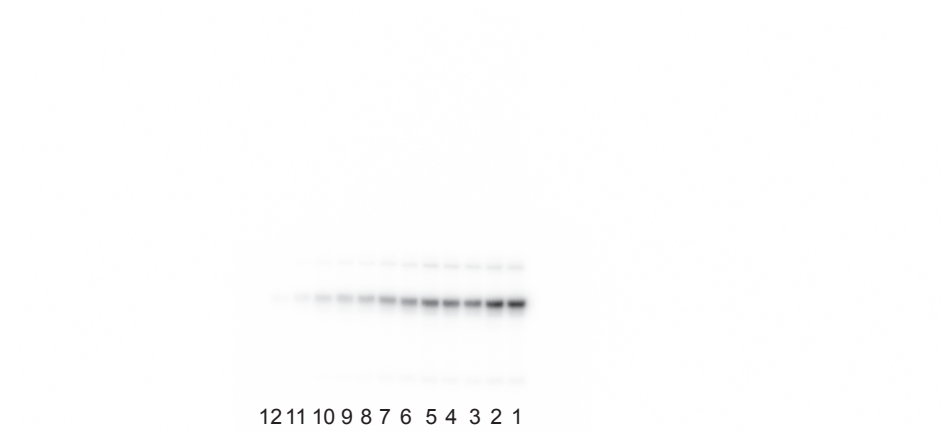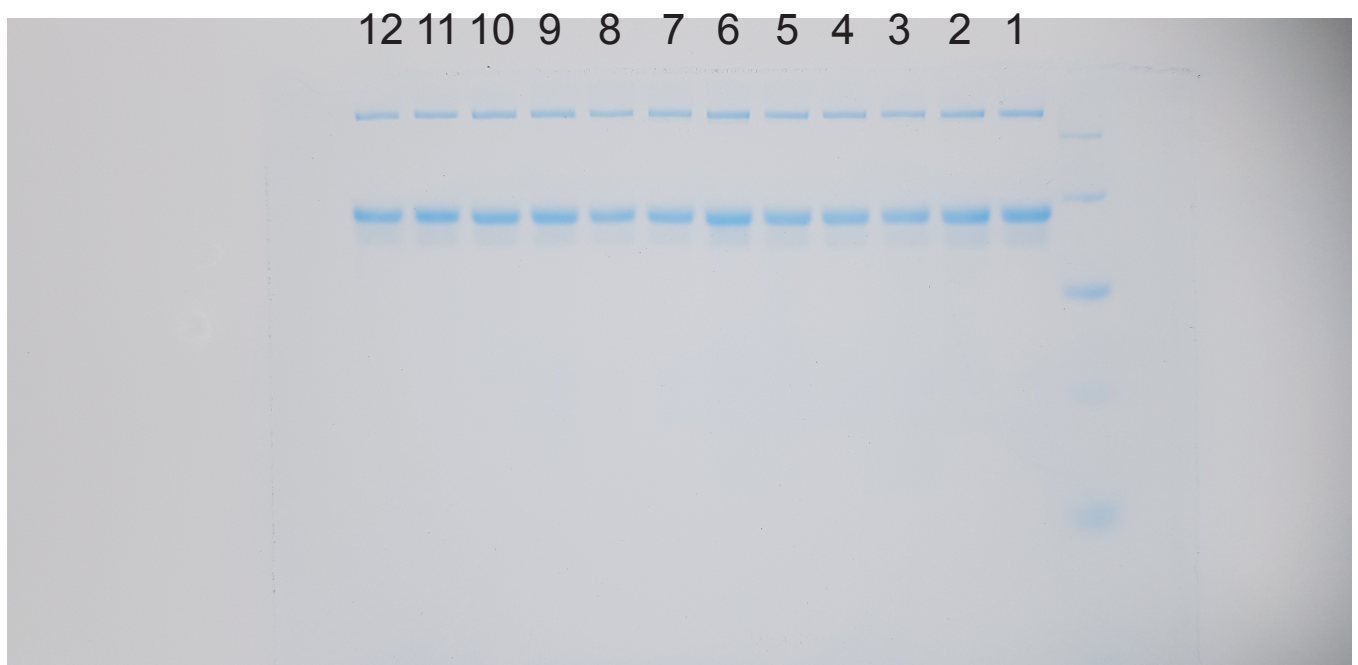

Supplement: Figure 1—source data 1. [file elife-72353-fig1-data1.zip › figure_1_source_data/source_fig1.pdf]

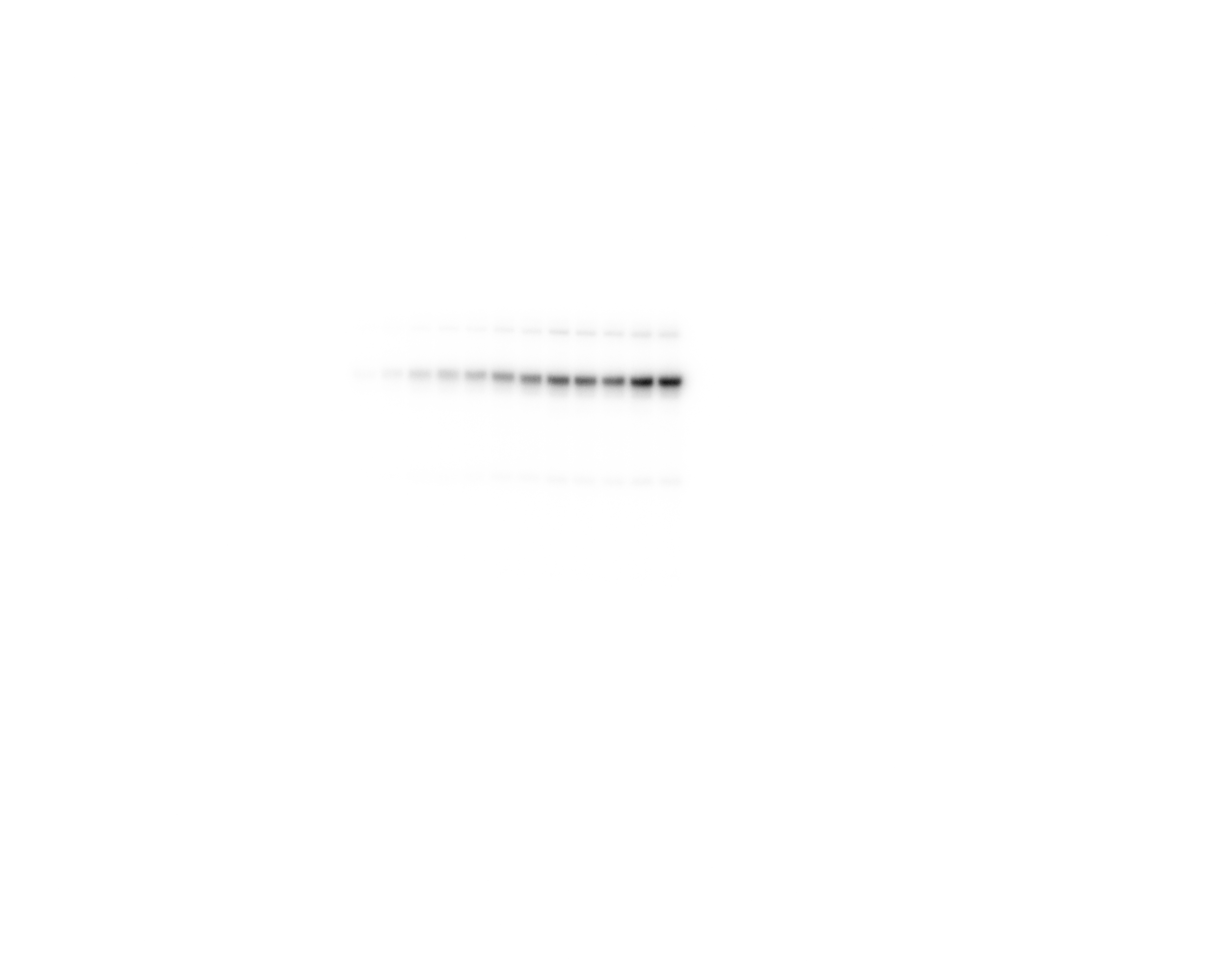

Supplement: Figure 1—source data 1. [file elife-72353-fig1-data1.zip › figure_1_source_data/fig_1.tif]

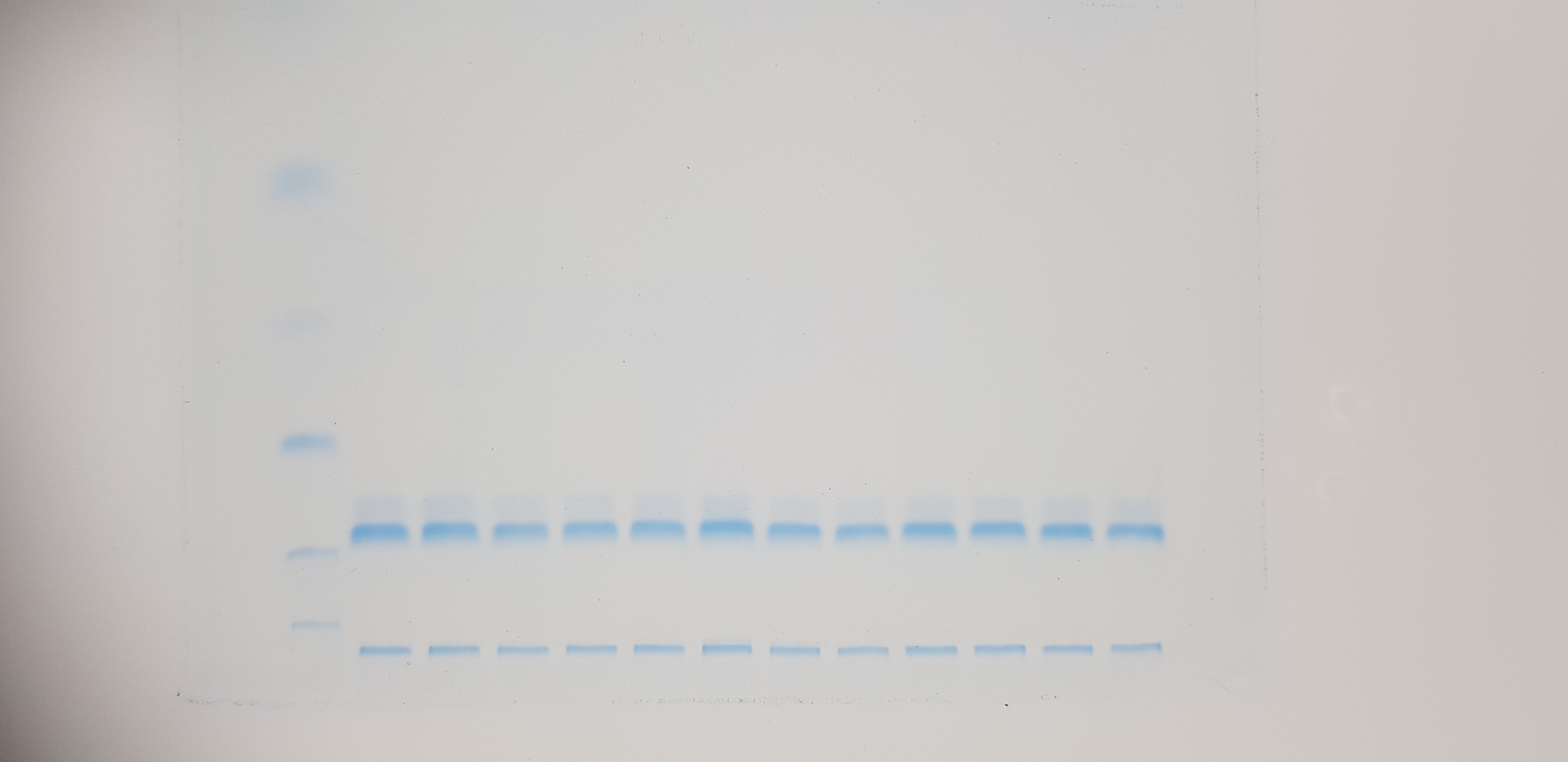

Supplement: Figure 1—source data 1. [file elife-72353-fig1-data1.zip › figure_1_source_data/fig_1.jpg]

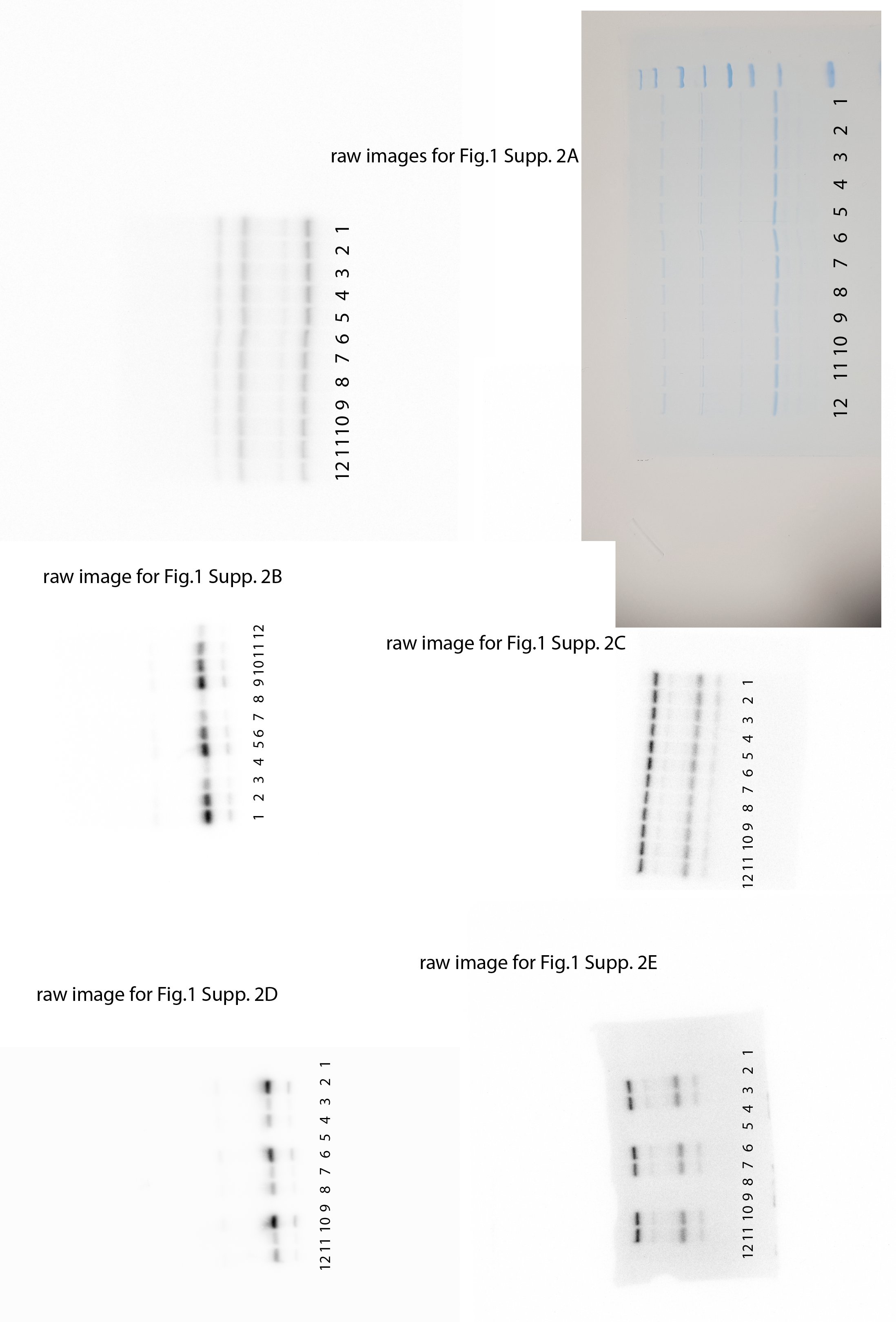

Supplement: Figure 1—figure supplement 2—source data 1. [file elife-72353-fig1-figsupp2-data1.zip › figure_1_supp2_source_data/source_data_edited.png]

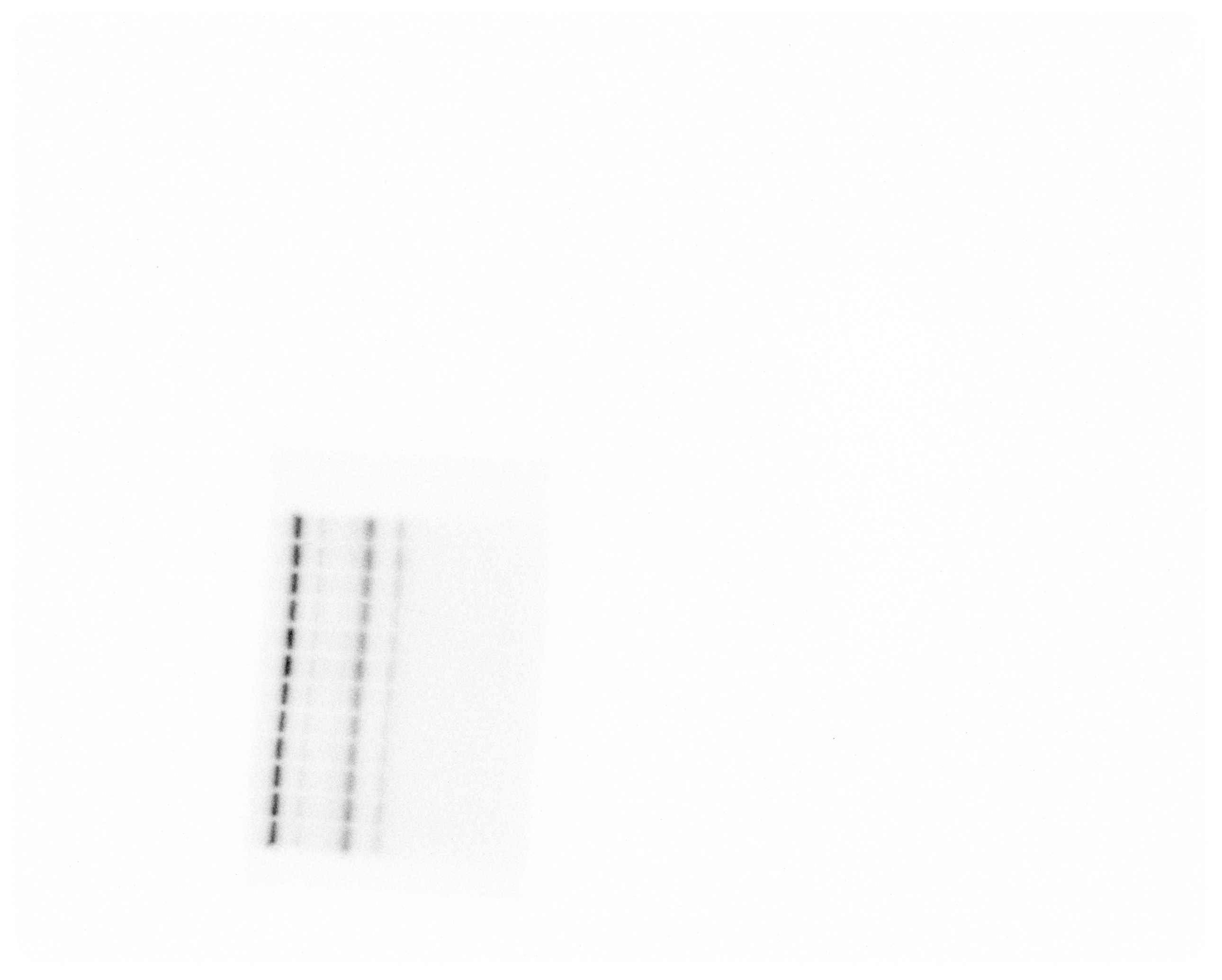

Supplement: Figure 1—figure supplement 2—source data 1. [file elife-72353-fig1-figsupp2-data1.zip › figure_1_supp2_source_data/panel_C/fig1_supp2_C.tif]

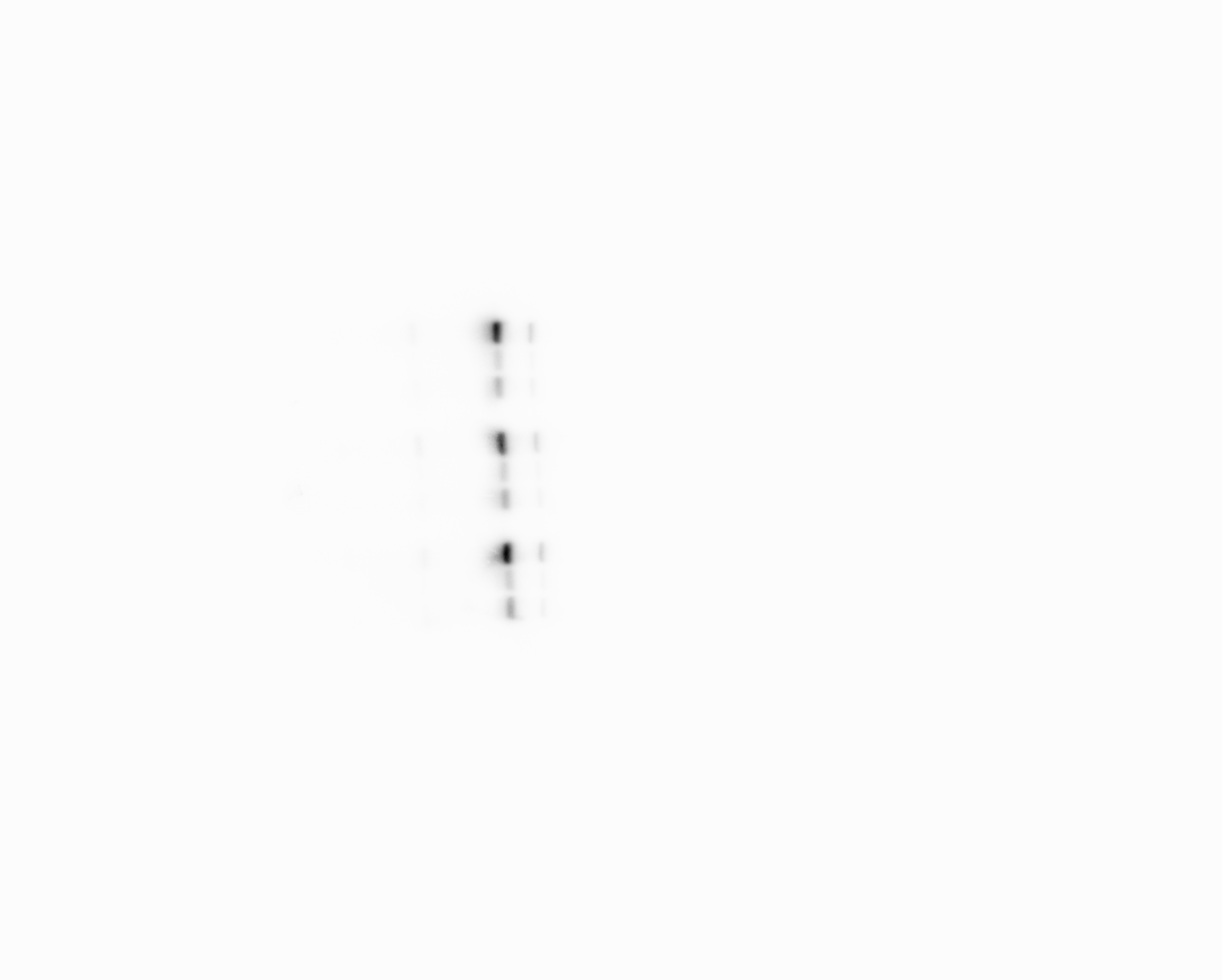

Supplement: Figure 1—figure supplement 2—source data 1. [file elife-72353-fig1-figsupp2-data1.zip › figure_1_supp2_source_data/panel_D/fig1_supp2_D.tif]

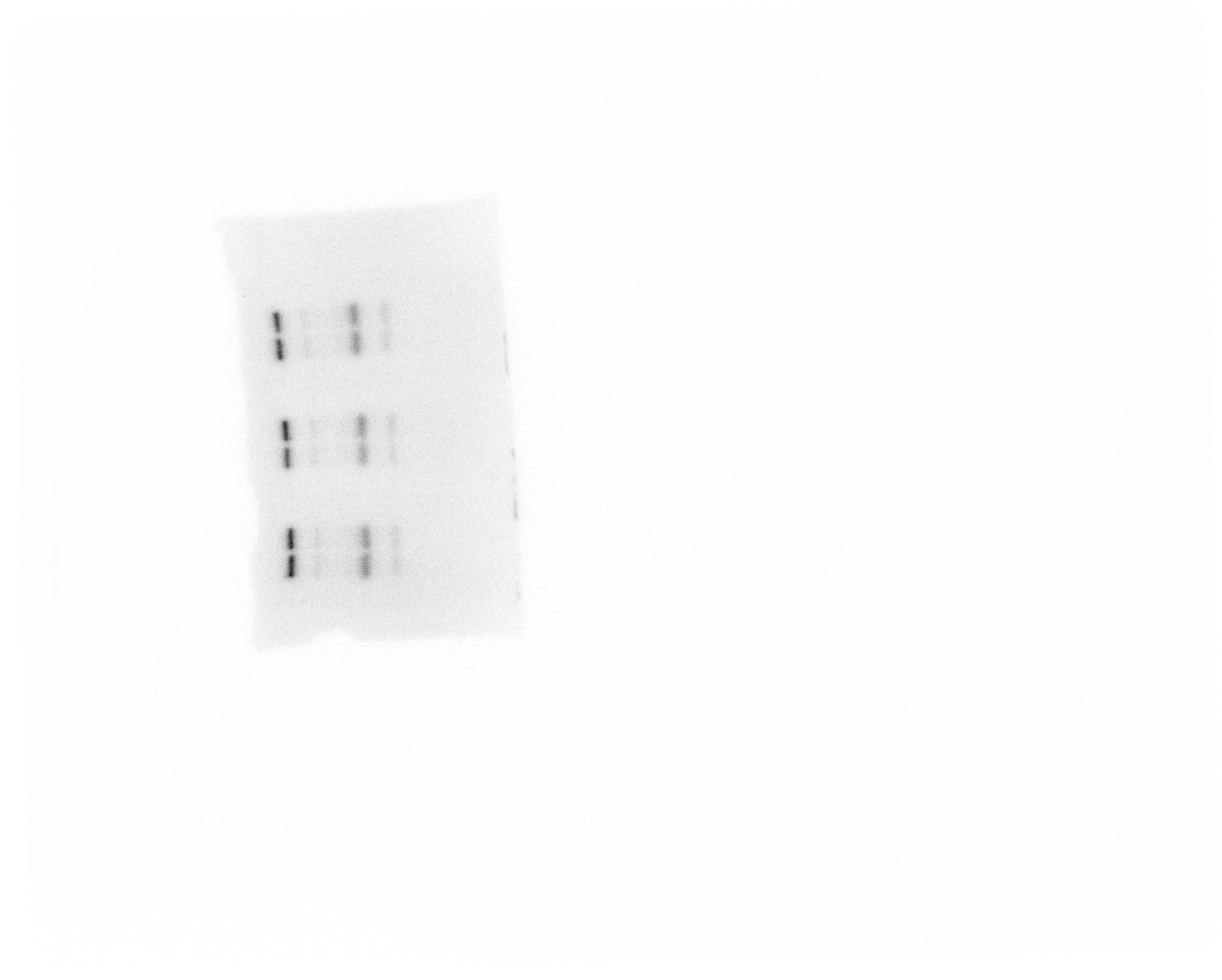

Supplement: Figure 1—figure supplement 2—source data 1. [file elife-72353-fig1-figsupp2-data1.zip › figure_1_supp2_source_data/panel_E/fig1_supp2_E.tif]

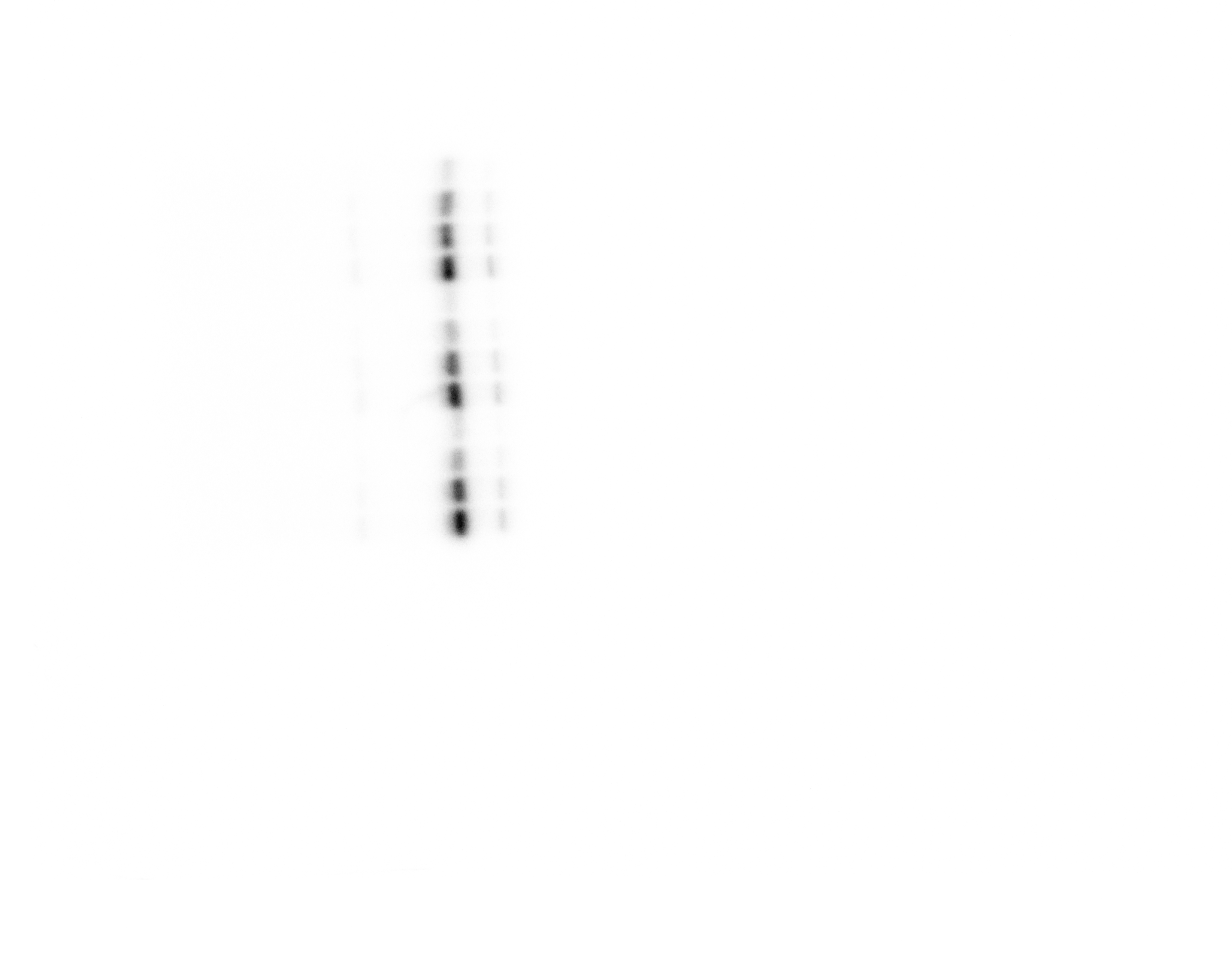

Supplement: Figure 1—figure supplement 2—source data 1. [file elife-72353-fig1-figsupp2-data1.zip › figure_1_supp2_source_data/panel_B/fig_1_supp2_B.tif]

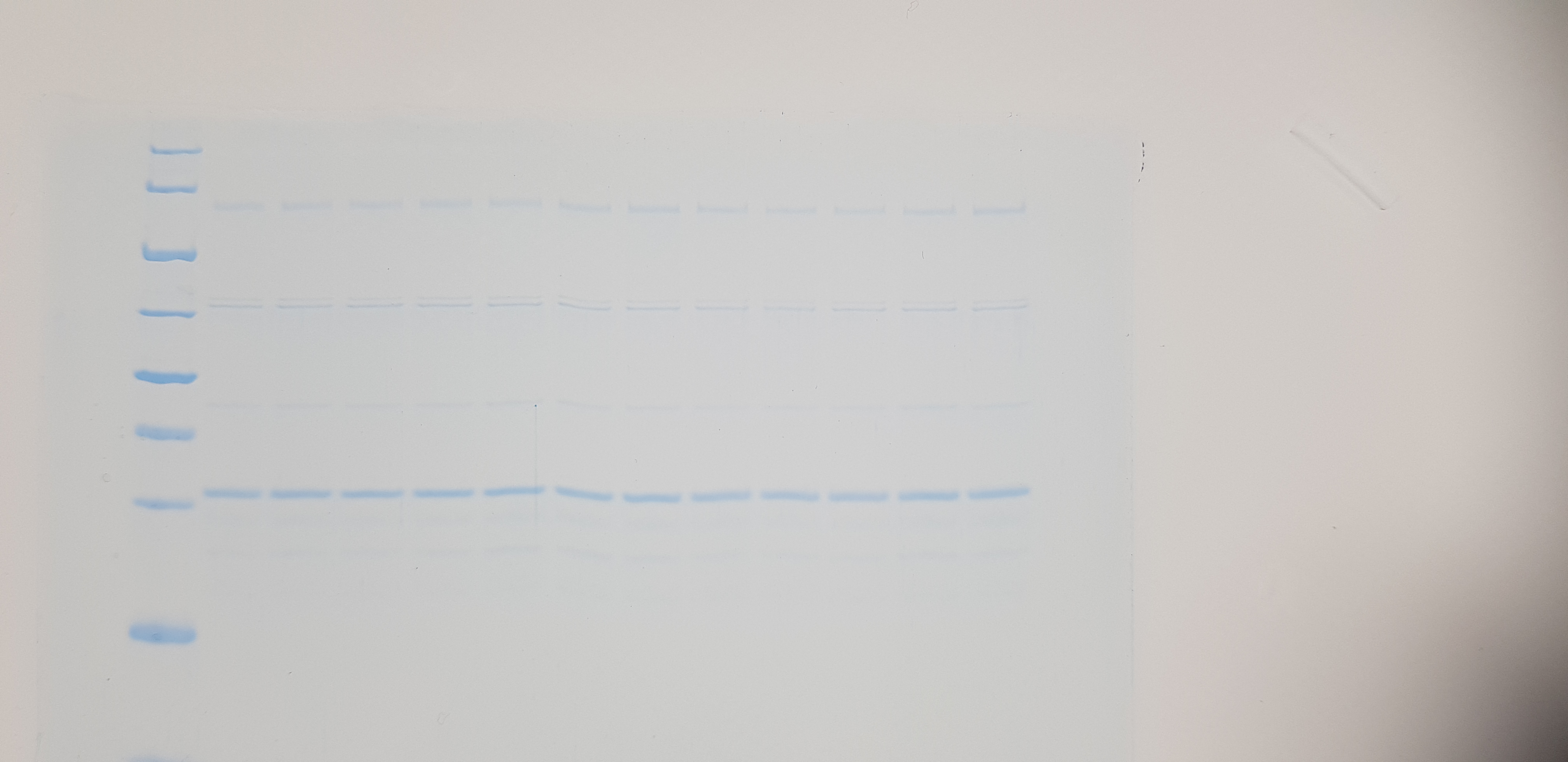

Supplement: Figure 1—figure supplement 2—source data 1. [file elife-72353-fig1-figsupp2-data1.zip › figure_1_supp2_source_data/panel_A/fig_1_supp2_A.jpg]

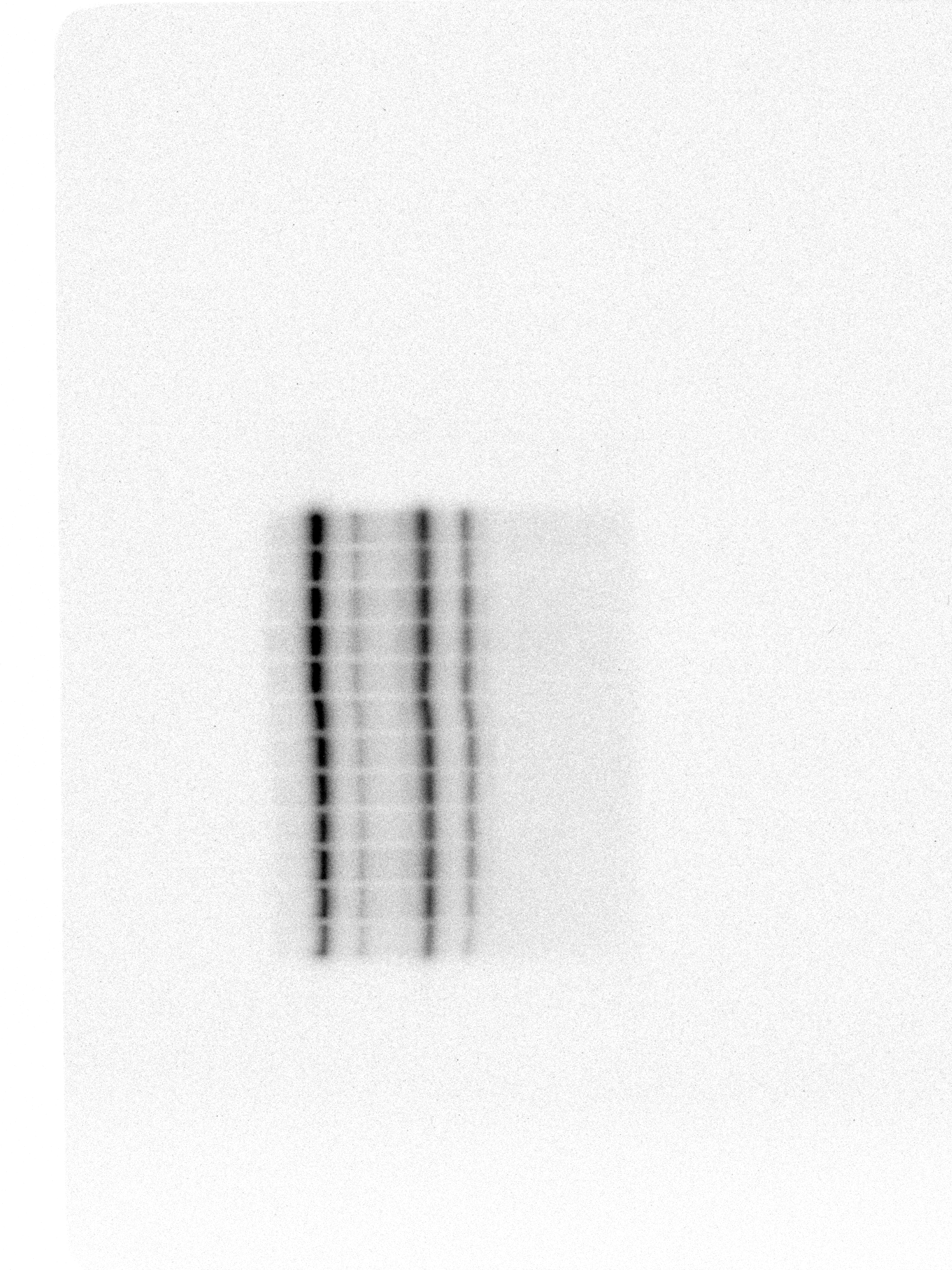

Supplement: Figure 1—figure supplement 2—source data 1. [file elife-72353-fig1-figsupp2-data1.zip › figure_1_supp2_source_data/panel_A/fig_1_supp2_A.tif]

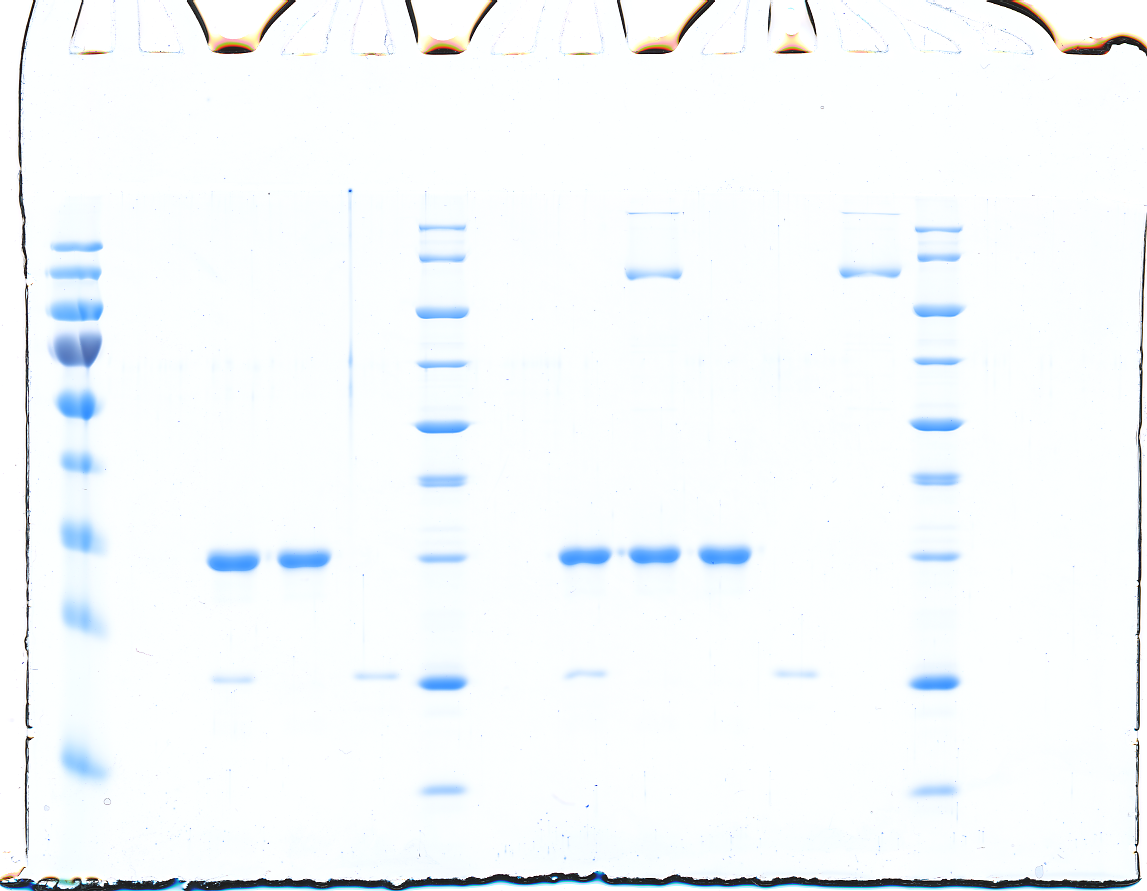

Supplement: Figure 4—source data 2. [file elife-72353-fig4-data2.zip › figure_4_source_data/Fig_4_In.tif]

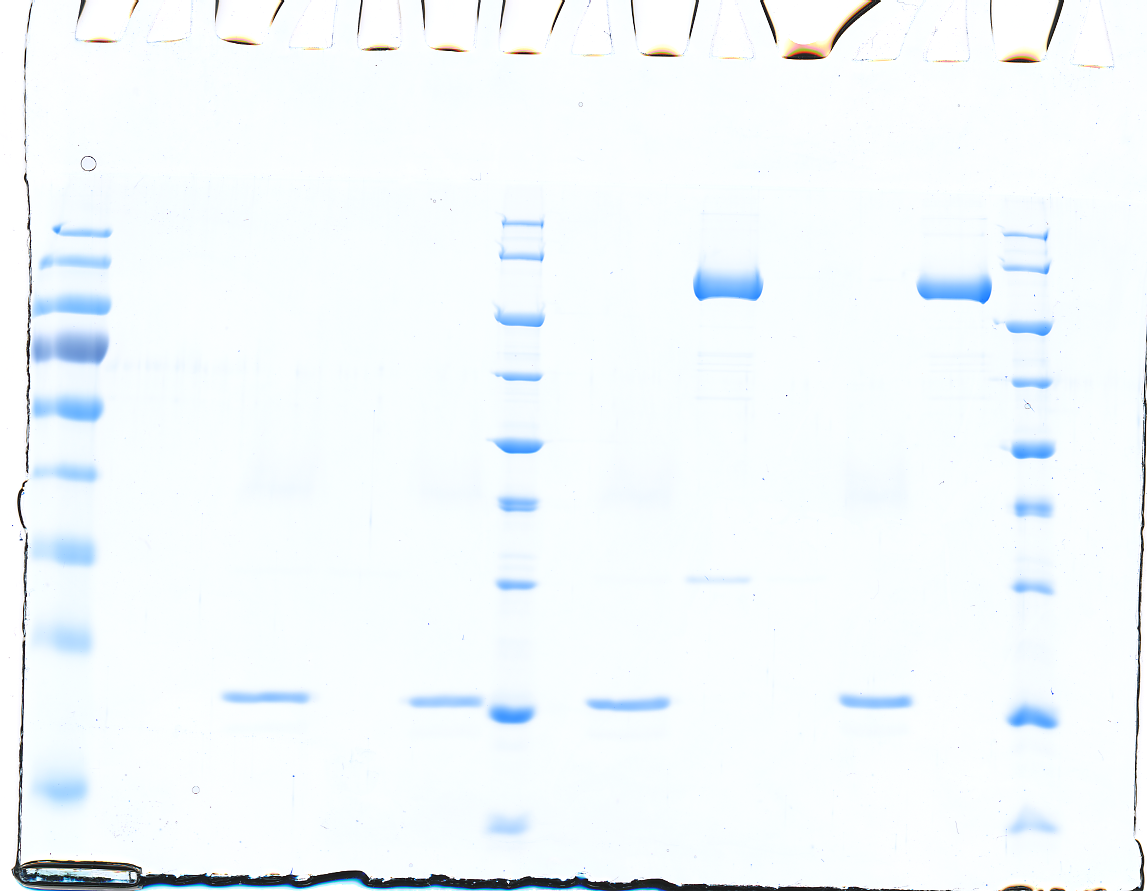

Supplement: Figure 4—source data 2. [file elife-72353-fig4-data2.zip › figure_4_source_data/Fig_4_E.tif]

raw images for Figure 4 D

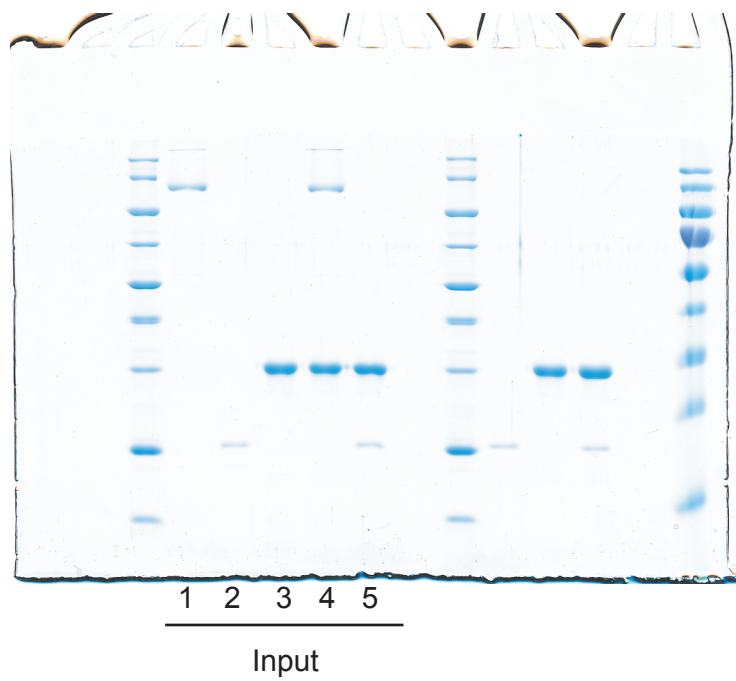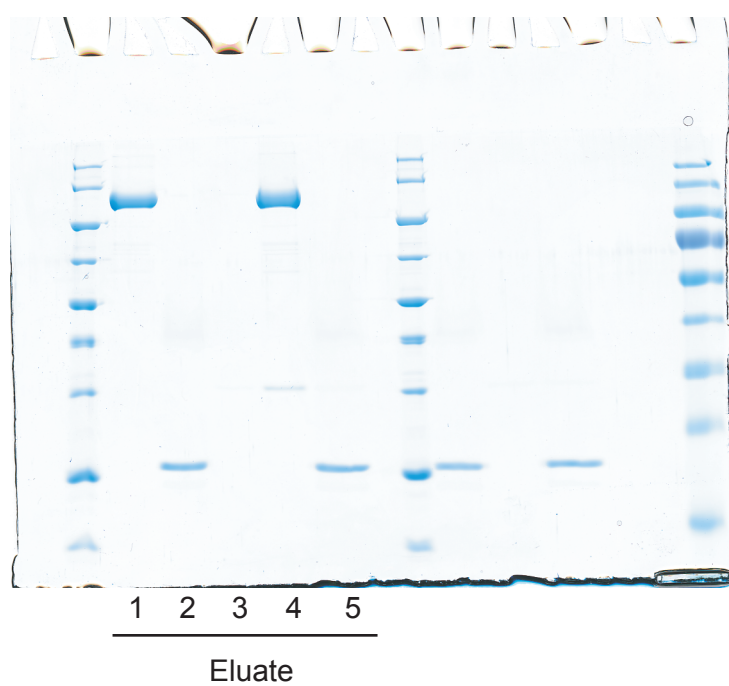

Supplement: Figure 4—source data 2. [file elife-72353-fig4-data2.zip › figure_4_source_data/source_fig4.pdf]

raw image for Figure 4 Supp. 1

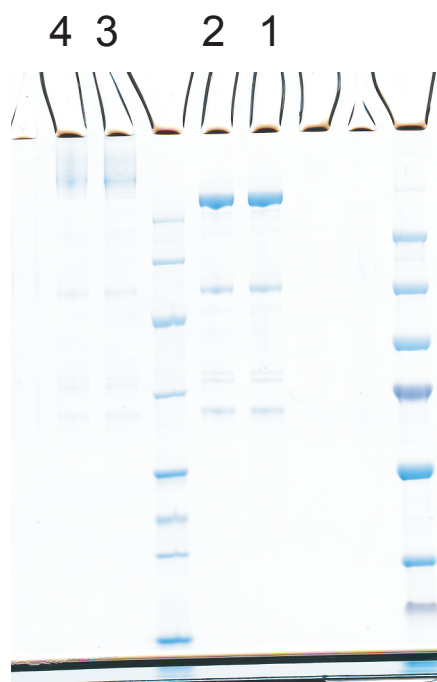

Supplement: Figure 4—figure supplement 1—source data 1. [file elife-72353-fig4-figsupp1-data1.zip › figure_4_supp1_source_data/source_fig4_supp1.pdf]

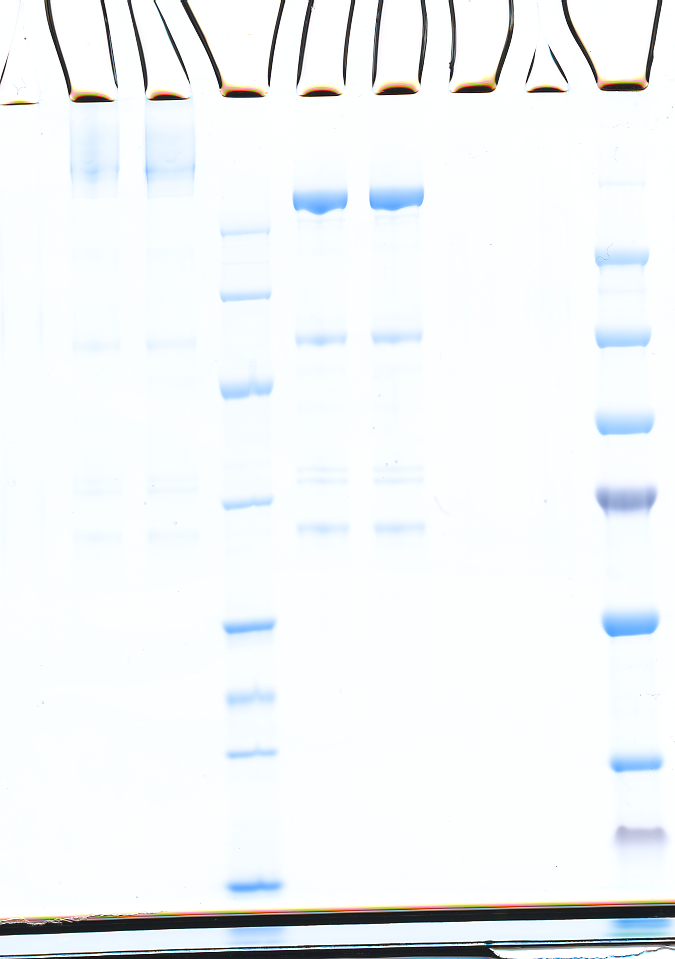

Supplement: Figure 4—figure supplement 1—source data 1. [file elife-72353-fig4-figsupp1-data1.zip › figure_4_supp1_source_data/fig_4_supp1.tif]

raw images for Figure 4 Supp. 3 A

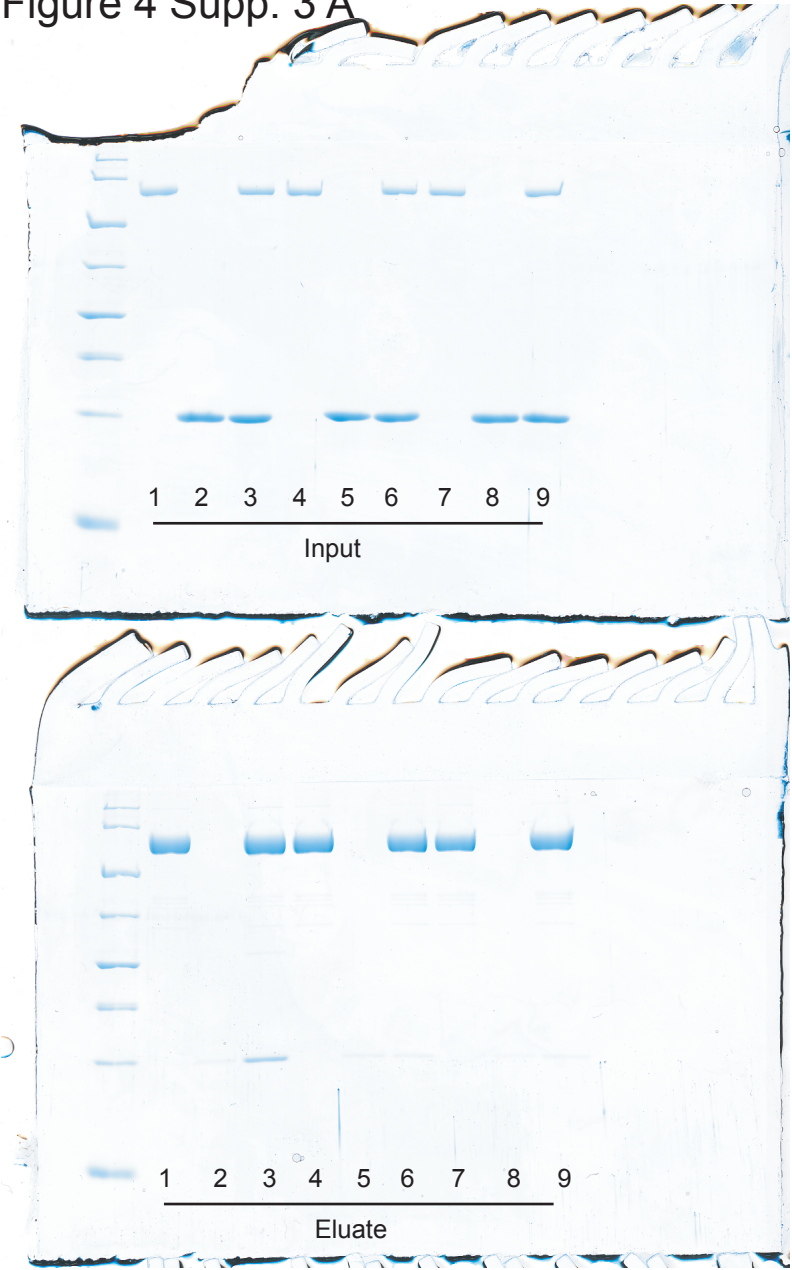

raw image for Figure 4 Supp. 3 B

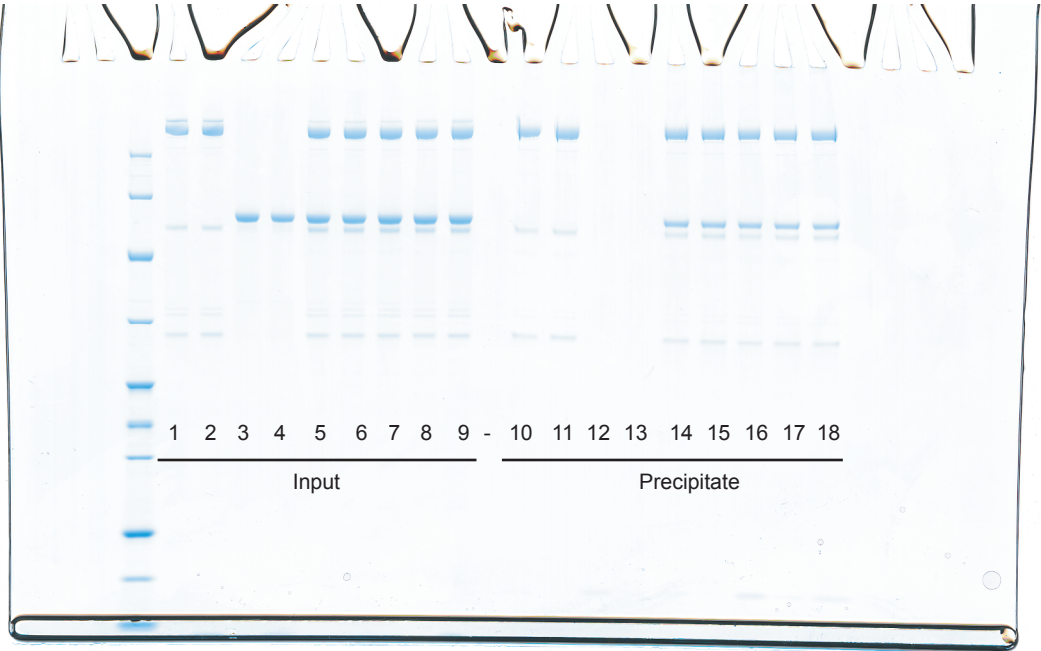

Supplement: Figure 4—figure supplement 4—source data 1. [file elife-72353-fig4-figsupp4-data1.zip › figure_4_supp4_source_data/source_fig4_supp4.pdf]

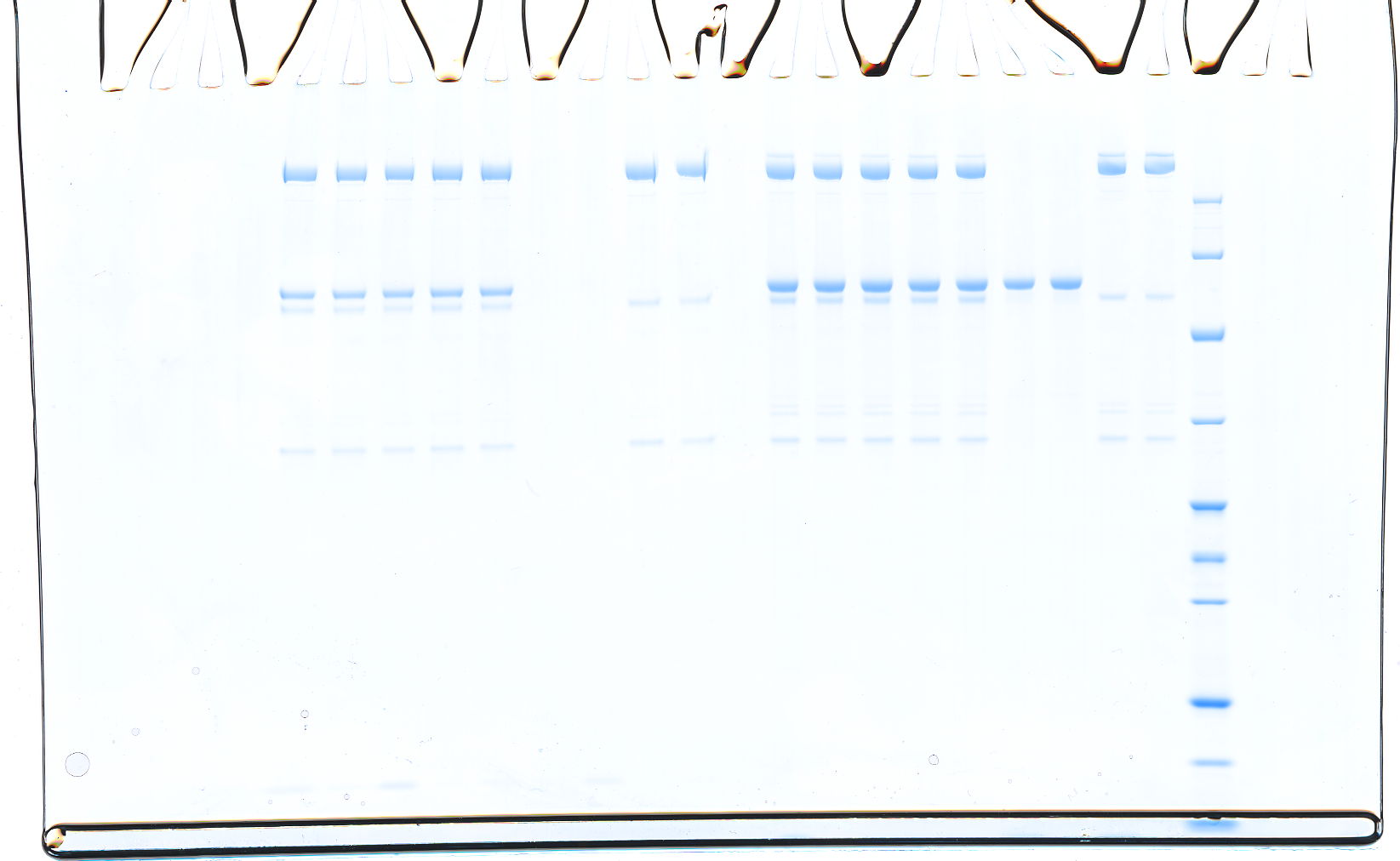

Supplement: Figure 4—figure supplement 4—source data 1. [file elife-72353-fig4-figsupp4-data1.zip › figure_4_supp4_source_data/panel_B/Fig_4_supp4_B.tif]

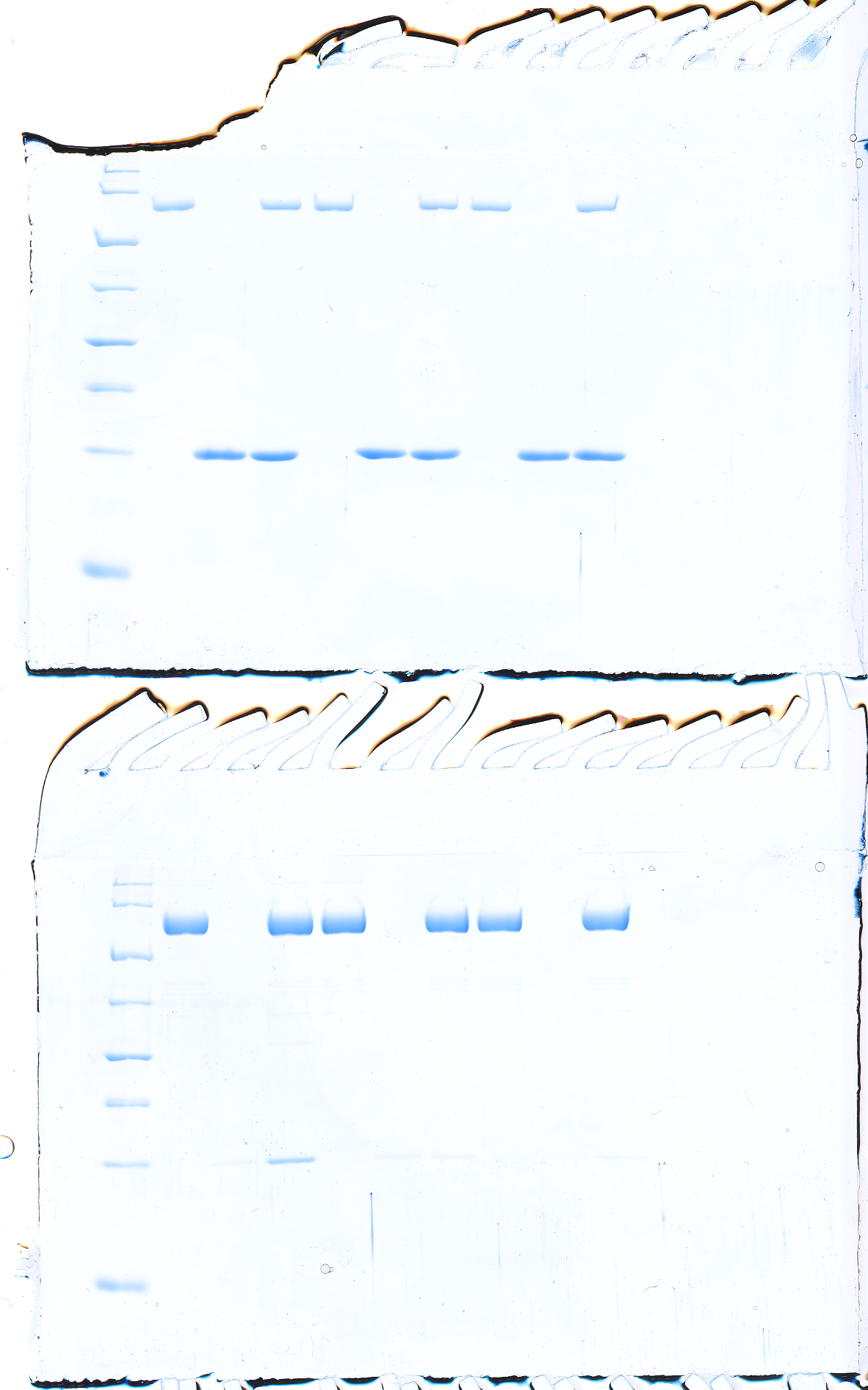

Supplement: Figure 4—figure supplement 4—source data 1. [file elife-72353-fig4-figsupp4-data1.zip › figure_4_supp4_source_data/panel_A/fig_4_supp4_A.tif]
