# Supplementary material for: Cryo-EM reconstructions of inhibitor-bound SMG1 kinase reveal an autoinhibitory state dependent on SMG8
Source: eLife. 2021 Oct 26;10:e72353. doi: 10.7554/eLife.72353 (PMC8592573; doi:10.7554/eLife.72353)
Supplement: Supplementary file 1. [file elife-72353-supp1.docx]

**Supplemental Table 1:** Cryo-EM data collection, refinement and validation statistics.

|  | SMG1 body + SMG1i | SMG1-8-9 + SMG1i | | SMG1-9 +  SMG1i | SMG1-8-9 + AMPPNP | SMG1-9 + AMPPNP |
| --- | --- | --- | --- | --- | --- | --- |
| Data availability |  |  |  | |  |  |
| EMDB | 13676 | 13674/  (13675) | 13677 | | 13678 | 13679 |
| PDB | 7PW6 | 7PW4/  (7PW5) | 7PW7 | | 7PW8 | 7PW9 |
| Data collection and processing |  |  |  | |  |  |
| Microscope |  |  | FEI Titan Krios GII | |  |  |
| Voltage (kV) |  |  | 300 | |  |  |
| Camera |  |  | Gatan K3 | |  |  |
| Energy filter |  |  | Gatan Quantum-LS (GIF) | |  |  |
| Magnification |  |  | 105,000x | |  |  |
| Pixel size (Å/pix) |  |  | 0.8512 | |  |  |
| Target defocus range (μm) |  |  | -0.5 to -2.5 | |  |  |
| electron exposure (e^-^/Å^2^) | 89.32 | 89.32 | 89.32 | | 60.99 | 60.99 |
| Number of movies | 8,784 | 8,784 | 8,784 | | 12,117 | 12,117 |
| Initially selected particle candidates | 1,898,472 | 1,898,472 | 1,898,472 | | 2,359,962 | 2,359,962 |
| Final number of particles | 228,291 | 228,291 | 85,216 | | 403,079 | 168,050 |
| Resolution _FSC independent halfmaps_ (Å)^a^ | 3.1 | 3.3 | 3.6 | | 2.8 | 3.1 |
| Refinement |  |  |  | |  |  |
| Initial model used | 6Z3R | 6Z3R | 6Z3R | | 6Z3R | 6Z3R |
| No. atoms | 10,396 | 18,973 | 16230 | | 18,965 | 16342 |
| Residues | 1416 | 2630 | 2251 | | 2629 | 2261 |
| Ligands |  |  |  | |  |  |
| SMG1i | 1 | 1 | 1 | | - | - |
| AMPPNP | - | - | - | | 1 | 1 |
| ATP | 1 | 1 | 1 | | 1 | 1 |
| IP6 | 1 | 1 | 1 | | 1 | 1 |
| Mg | 1 | 1 | 1 | | 1 | 1 |
| CC_mask,_ CC_box_, CC_peaks_, CC_volume_^b^ | 0.82, 0.79, 0.73, 0.79 | 0.82, 0.79, 0.71, 0.80 | 0.76, 0.76,  0.65, 0.73 | | 0.82, 0.78, 0.75, 0.79 | 0.79, 0.73, 0.67, 0.76 |
| Resolution _FSC map vs. model (0/0.143/0.5)_ (Å)^b^ | 2.5/2.9/3.1 | 3.0/3.0/3.3 | 3.3/3.4/3.8 | | 2.1/2.5/2.7 | 2.9/3.0/3.3 |
| *B* factors ( Å^2^) |  |  |  | |  |  |
| Protein | 21.99 | 80.93 | 71.54 | | 20.68 | 44.18 |
| Ligands | 31.88 | 72.59 | 67.59 | | 61.23 | 37.92 |
| RMs deviations |  |  |  | |  |  |
| Bond lengths (Å) | 0.002 | 0.002 | 0.002 | | 0.002 | 0.002 |
| Bond angles (°) | 0.543 | 0.529 | 0.527 | | 0.631 | 0.562 |
| Validation |  |  |  | |  |  |
| Ramachandran plot |  |  |  | |  |  |
| Favored (%) | 97.28 | 97.42 | 97.27 | | 96.38 | 96.97 |
| Allowed (%) | 2.72 | 2.58 | 2.73 | | 3.17 | 2.99 |
| Disallowed (%) | 0.00 | 0.00 | 0.00 | | 0.00 | 0.05 |
| MolProbity score | 1.33 | 1.34 | 1.41 | | 1.38 | 1.45 |
| Clash score | 4.17 | 4.45 | 5.19 | | 4.02 | 5.15 |
| Rotamer outliers (%) | 0.10 | 0.00 | 0.00 | | 0.06 | 0.00 |
|  |  |  |  | |  |  |

**^a^**according to the Fourier Shell Correlation (FSC) cut-off criterion of 0.143 defined in (Rosenthal and Henderson, 2003)

**^b^**according to the map-vs.-model Correlation Coefficient definitions in (Afonine et al., 2018a)

**References**

Rosenthal, P. B., and R. Henderson. 2003. 'Optimal determination of particle orientation, absolute hand, and contrast loss in single-particle electron cryomicroscopy', *J Mol Biol*, 333: 721-45.

Tan, Y. Z., P. R. Baldwin, J. H. Davis, J. R. Williamson, C. S. Potter, B. Carragher, and D. Lyumkis. 2017. 'Addressing preferred specimen orientation in single-particle cryo-EM through tilting', *Nat Methods*, 14: 793-96.
